# Niche Distribution Pattern of Rüppell's Vulture (*Gyps rueppellii*) and Conservation Implication in Kenya

**DOI:** 10.1002/ece3.70371

**Published:** 2024-12-06

**Authors:** Purity Chepkirui, David Chiawo, Jumbe James, Simbauni Jemimah, Elizabeth Ellwood, Jane Mugo, Peggy Mutheu Ngila

**Affiliations:** ^1^ Department of Zoological Sciences Kenyatta University Nairobi Kenya; ^2^ Centre for Biodiversity Information Development (BID‐C) Strathmore University Nairobi Kenya; ^3^ IDigBio University of Florida Gainesville Florida USA; ^4^ Department of Earth and Climate Science University of Nairobi Nairobi Kenya

**Keywords:** critically endangered species, habitat suitability, Rüppell's vultures, species distribution modeling

## Abstract

Rüppell's vultures are critically endangered, primarily due to anthropogenic activities such as habitat degradation, climate change, and intentional and unintentional poisoning, which have led to the loss of nesting and breeding sites. To aid in the conservation and protection of these species, habitat evaluation and niche mapping are crucial. Species distribution modeling (SDM) is a valuable tool in conservation planning, providing insights into the ecological requirements of species under conservation concerns. This study employed an ensembling modeling approach to assess the habitat suitability and distribution of Rüppell's vultures across Kenya. We utilized four algorithms; Gradient Boosting Machine, Generalized Linear Model, Generalized Additive Model, and Random Forest. Data on Rüppell's vultures were sourced from the Global Biodiversity Information Facility, while key environmental variables influencing the species' distribution were obtained from WorldClim. The resultant species distribution map was overlaid with a conservation area map to evaluate the overlap between suitable habitats and existing protected areas. Our analysis identified suitable habitats in regions such as the Masai Mara Game Reserve, Mount Kenya National Park, Nairobi National Park, Tsavo East National Park, and Hell's Gate National Park, with the majority of these habitats located outside protected areas, except those within Hell's Gate National Park. Precipitation and elevation emerged as the primary environmental predictors of the distribution of Rüppell's vultures. Based on these findings, we recommend establishing vulture sanctuaries in suitable habitats and hotspots to enhance the conservation of Rüppell's vultures outside the protected areas.

## Graphical abstract

The study is showing the habitat distribution of Ruppell's vultures in Kenya.

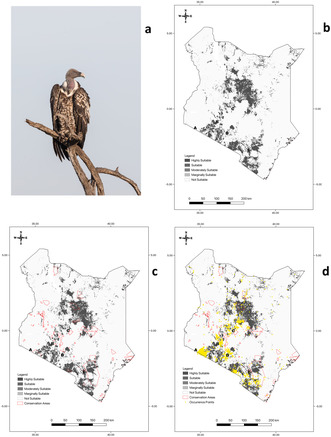

## 1 Introduction

Rüppell's vultures are critically endangered primarily due to anthropogenic activities like habitat degradation, climate change, and intentional and unintentional poisoning. The decline of vulture populations particularly in Africa has been a matter of great concern as evidenced by various studies (Buechley and Şekercioğlu 2016; Krüger et al. 2014; Ogada, Keesing, and Virani 2012; Ogada, Botha, and Shaw 2016; Ogada, Shaw, et al. 2016; Ogada and Buij 2011) and has underscored the significant reduction in vulture numbers. Vultures hold immense ecological importance due to their rapid carrion consumption, effective control of problematic scavenger species, and potential for disease regulation (Ogada, Keesing, and Virani 2012). As a consequence of the heightened vulnerability, conservation efforts have distinctly prioritized their preservation (Buechley and Şekercioğlu 2016; Plaza, Blanco, and Lambertucci 2020). Within the spectrum of vulture species, encompassing eight *Gyps* varieties globally, Kenya is host to two resident species (*G. africanus* and *G. rueppellii*) while the remaining six are migratory (*G. bengalensis*, *G. fulvus*, *G. coprotheres*, *G. tenuirostris*, *G. indicus*, and *G. himalayensis*). These two resident *Gyps* vulture species, *G. africanus* and *G. rueppellii*, are critically endangered as a result of various threats including habitat transformation into agro‐pastoral systems, loss of wild ungulates leading to reduced availability of carrion, illicit trade, persecution, and deliberate poisoning (Ogada, Botha, and Shaw 2016; Ogada, Shaw, et al. 2016).

Reports of regional reductions of vultures have also been reported in West Africa (Thiollay 2006), southern Africa (Bamford et al. 2007; Boshoff, Piper, and Michael 2009), and East Africa (Ogada and Keesing 2010; Virani et al. 2011; Ogada, Keesing, and Virani 2012). In Kenya, for instance, some vulture species are now classified as “vulnerable”, while others are listed as “near threatened”, due to ongoing population declines (Khatri 2015). This continuous downward trajectory in vulture numbers raises profound concerns about the stability of these species and underscores the urgency for comprehensive conservation strategies to mitigate their declining numbers.

The distribution of Rüppell's vulture spans across the Sahel region and East Africa, occupying diverse habitats such as Acacia woodlands, grasslands, and montane regions. Displaying a gregarious nature, this species congregates at carrion sites, engaging in collective soaring within flocks. Its breeding behavior predominantly takes place in colonies situated on cliff faces and escarpments, encompassing a wide range of elevations. Notably, Rüppell's vulture finds its breeding and nesting havens among cliffs in both northern and southern Kenya, as well as in Tanzania. These sites serve as focal points where substantial populations of Rüppell's vultures congregate, engaging in the nurturing of offspring and foraging activities within the surrounding vicinity (Virani et al. 2012). In Kenya, significant breeding locations for Rüppell's vultures encompass Kichwa Tembo, Soit Pus, Kwenia, Hell's Gate, Ololokwe, and Losai (Virani et al. 2012). These sites play a pivotal role in the species' reproductive and ecological dynamics, making them essential focal points for conservation efforts aimed at safeguarding the future of Rüppell's vulture populations.

Understanding the distribution of species and the use of habitat is critical for determining spatial conservation priorities. Species distribution models (SDMs) are critical tools for forecasting climatic and anthropogenic effects on species and identifying priority habitats (Aryal et al. 2016; Guisan and Thuiller 2005). Species distribution models can assist in determining conservation priorities when combined with data on protected areas and current threats to species (De Barros et al. 2012). By linking the occurrence of a species at a given location to environmental features like topography (elevation, slope aspect, etc.) and bioclimatic factors like temperature and precipitation, a habitat suitability index (HSI) can be developed to establish the species niche range (Guisan and Thuiller 2005) to inform conservation planning. This study, therefore, applied species distribution modeling (SDM) to examine the spatial distribution of Rüppell's vulture in Kenya and the relative effect of environmental predictors on their habitat suitability. The findings are intended to raise awareness of distribution and inform the planning and conservation management of protected areas and suitable habitats for the conservation of Rüppell's vulture.

## 2 Materials and Methods

### 2.1 Study Area

The study focused on the distribution of Rüppell's vulture across Kenya's landmass, covering an area of 580,367 km^2^, located between latitudes 5°40′ N and 4°4′ S and longitudes 33°60′ E and 41°45′ E. The atmospheric temperature in Kenya ranges from 15°C to 35°C and has increased at an average rate of 0.21°C per decade since 1960 and is projected to increase by 1.6°C to 2.7°C by 2060s. The country has a tropical climate ranging from hot and humid along the coast to mild inland and exceptionally dry in the North and North‐East. Long rains start from April to June, while short rains start from October to December. February and March are the hottest months, while July and August are the coolest. Around 10% of Kenya's land mass is designated as protected areas, including national parks and reserves for wildlife conservation. The first of these was Nairobi National Park, which opened in 1946. Other National parks and reserves include; Masai Mara National Reserves, Mount Kenya National Park and Reserve, Tsavo East National Park, Hell's Gate National Park, and Amboseli National Park.

### 2.2 Data Preprocessing

A total of 96 occurrence records for Rüppell's vultures were obtained from the Global Biodiversity Information Facility (GBIF.org, 11 April 2024, GBIF Occurrence Download https://doi.org/10.15468/dl.wh4ee7). To ensure data accuracy and reliability, we used the ‘clean_coordinates’ wrapper function from the coordinate cleaner package in R (Zizka et al. 2018). This process addressed several inconsistencies, such as incomplete or zero coordinates, misaligned coordinates with the indicated country information, outliers, coordinates associated with biodiversity institutions, and those representing central points of countries or provinces. Additionally, urban areas were identified and excluded from the analysis. Furthermore, we implemented stringent criteria to ensure that only species‐level records directly relevant to the specific taxonomic group under investigation were included.

To address spatial autocorrelation and sampling bias in the occurrence data, we employed a spatial filtering technique. This approach was crucial in preventing model overfitting and improving the accuracy of our findings (Boria et al. 2014). To mitigate oversampling in regions with extensive surveys, we applied a spatial filter distance of 40 km, based on previous research on falcons and other raptors (Sutton et al. 2020). We used the “thin” algorithm function from the R package SpThin, to identify and remove clustered occurrence points (Aiello‐Lammens et al. 2015). After data cleaning, 94 (2 records removed) records were used for species distribution modeling.

### 2.3 Environmental Modeling Data

To perform ecological niche modeling to determine the geographic distribution of Rüppell's vultures in Kenya, 24 variables (raster layers) including bioclimatic and geographical variables were tested (Table 1). Nineteen bioclimatic variables with a spatial resolution of 30 arc‐seconds (roughly 1 km^2^) were downloaded from the World Climate Database (http://worldclim.org/bioclim) (Fick and Hijmans 2017) for the average period of 1970–2000 for climatic data.

**TABLE 1:** The Bioclimatic variables used to make the current vulture habitat projection.

Variables	Acronym	Units	Source
Isothermality	BIO3	Percentage (%)	http://worldclim.org/bioclim
Annual temperature range	BIO7	Degrees Celsius (°C)	http://worldclim.org/bioclim
Precipitation of the wettest month	BIO13	Millimeters (mm)	http://worldclim.org/bioclim
Precipitation of the driest month	BIO14	Millimeters (mm)	http://worldclim.org/bioclim
Precipitation of the warmest quarter	BIO18	Millimeters (mm)	http://worldclim.org/bioclim
Precipitation of the coldest quarter	BIO19	Millimeters (mm)	http://worldclim.org/bioclim
Geographical variables			
Aspect	Asp	Degree (°)	Digital Elevation Model in Worldclim
Human influence index	HII	Human influence index	https://sedac.ciesin.columbia.edu/data/collection/wildareas‐v2
Normalized difference vegetation index	NDVI	Normalized difference vegetation index	www.earthexplorer.usgs.gov
Elevation	Ele	Meters (m)	Digital Elevation Model in Worldclim
Slope	Slp	Degree (°)	Digital Elevation Model in Worldclim
Variables removed after the multicollinearity test			
Annual mean temperature	BIO1	Degrees Celsius (°C)	http://worldclim.org/bioclim
Annual mean diurnal range	BIO2	Degrees Celsius (°C)	http://worldclim.org/bioclim
Temperature seasonality	BIO4	Degrees Celsius (°C)	http://worldclim.org/bioclim
Max temperature of warmest month	BIO5	Degrees Celsius (°C)	http://worldclim.org/bioclim
Min temperature of coldest month	BIO6	Degrees Celsius (°C)	http://worldclim.org/bioclim
Mean temperature of wettest quarter	BIO8	Degree Celsius (°C)	http://worldclim.org/bioclim
Mean temperature of driest quarter	BIO9	Degree Celsius (°C)	http://worldclim.org/bioclim
Mean temperature of warmest quarter	BIO10	Degree Celsius (°C)	http://worldclim.org/bioclim
Mean temperature of coldest quarter	BIO11	Degree Celsius (°C)	http://worldclim.org/bioclim
Annual precipitation	BIO12	Millimeters (mm)	http://worldclim.org/bioclim
Precipitation seasonality	BIO15	Millimeters (mm)	http://worldclim.org/bioclim
Precipitation of driest quarter	BIO17	Millimeters (mm)	http://worldclim.org/bioclim

A multicollinearity test was performed using USDM version 1.1‐18 R package (Naimi et al. 2014), and variance inflation factor (VIF < 3) was used to get rid of highly correlated environmental predictors among the 24 variables (Ngila et al. 2023; Zuur, Ieno, and Elphick 2010). This was to ensure that only environmental variables with ecological relevance were used in the model. When the environmental elements used to train the model are significantly correlated, it might be difficult to interpret the model's output (Ngila et al. 2023; Phillips, Anderson, and Schapire 2006), particularly the relative significance of the variables and their response curves. After removing collinearity, 11 variables remained and were used to generate the model (Table 1).

### 2.4 Species Distribution Modeling

We utilized a combination of four robust models: General boosted regression model (GBM), General additive model (GAM), General linear model (GLM), and Random Forest (RF) to analyze the impact of bioclimatic and geographical variables on the occurrence of Rüppell's Vultures in Kenya. These models were run using an ensemble approach using the “biomod2” package in R (Ngila et al. 2023; Smeraldo et al. 2020). GLMs were configured with a quadratic link function and a maximum interaction level set to one. GBMs were constructed with a maximum of 5000 trees. GAMs employed a binomial link function, while RF models consisted of 750 trees, with half of the available predictors sampled for splitting at each node (Thuiller et al. 2009).

The occurrence dataset was split into two subsets: 70% for model calibration and 30% for model evaluation (Smeraldo et al. 2020). To compensate for the lack of absence data in our dataset, we randomly generated 10,000 pseudoabsences. Given the broad geographical scope of our study, encompassing all of Kenya, this number was considered appropriate (Barbet‐Massin et al. 2012; Phillips, Anderson, and Schapire 2006). The data‐splitting process was repeated twice, and the evaluation metrics were averaged across both iterations. In total, 40 species distribution models (SDMs) were generated, comprising four algorithms run five times each, with two replicates for model evaluation.

### 2.5 Model Testing

The accuracy of the final models was measured using the area under the Areas under the Receiver Operating Characteristic (ROC) curve and True Skill Statistics (TSS) (Bosch et al. 2014; Stockwell and Peters 1999). AUC values closer to 1 indicate higher model accuracy, while values near 0.5 suggest the model performs no better than chance (Bosch et al. 2014). AUC values are categorized as follows: > 0.9 = excellent, 0.7–0.9 = good, and 0.7 = uninformative (Baldwin 2009; Lv et al. 2012; Swets 1988). TSS values are classified as follows: < 0.40 = poor, 0.40–0.60 = fair, 0.60–0.80 = good, and 0.80–1.0 = excellent (Rew et al. 2020). Additionally, the importance of variables for the species was determined from the ensemble prediction. Model performance was evaluated using four accuracy metrics: TSS, AUC, specificity, and sensitivity (Bosch et al. 2014).

### 2.6 Reclassification of the Species Distribution

The examined simulated ensemble outputs were able to forecast habitat appropriateness as well as the geographic region currently occupied by Rüppell's Vultures. Using equal natural breaks from 0 to 1, the habitat appropriateness was categorized into five equally sized groups. They were arbitrarily regrouped as highly suitable (> 0.8) which is a land with optimal conditions suitable for Rüppell's Vultures, suitable (0.6–0.8) which is lands with minor climatic limitations for optimal Rüppell's Vultures, moderately suitable (0.4–0.6) which is lands with more minor climatic limitations for Rüppell's Vultures, marginally suitable (0.2–0.4) that is land with major climatic limitations that may significantly reduce the number of Rüppell's Vultures, and Unsuitable (< 0.2) Lands with severe climatic limitations that are not favorable for the survival of Rüppell's Vultures.

### 2.7 Overlap of Distribution of Rüppell's Vultures With Conservation Areas in Kenya

A shapefile of the conservation areas in Kenya was obtained from https://datasets.wri.org/dataset/protected‐areas‐in‐kenya and overlaid on the species distribution map of Rüppell's Vultures to establish the overlap between the niche range and the geographical boundaries of the conservation areas.

## 3 Results

### 3.1 Suitability Zones for Rüppell's Vulture Survival

**FIGURE 1:**
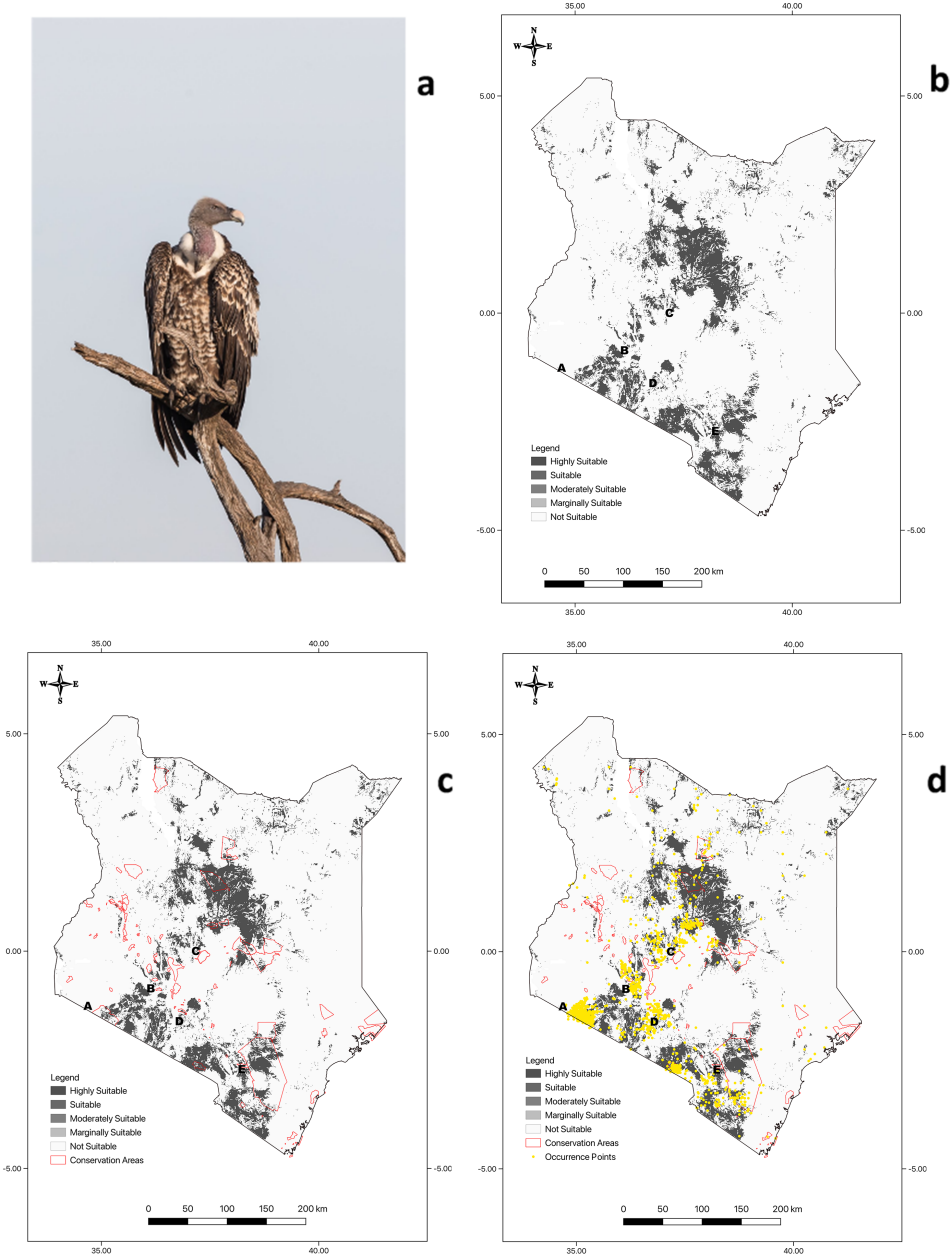
(a) Rüppell's Vulture observed in Tanzania by Greg Lasley (licensed under http://creativecommons.org/licenses/by‐nc/4.0/); (b) Map of Kenya showing the niche distribution and habitat suitability of Rüppell's' vulture; (c) Rüppell's Vulture occurrence and niche overlap with protected areas in Kenya (A = Masai Mara National Reserve, B = Hell's Gate National Park, C = Mount Kenya National Park, D = Nairobi National Park, and E = Tsavo East National Park); (d) Rüppell's vulture occurrence density in association with the protected areas in Kenya.

A habitat suitability map (Figure 1b) was developed to illustrate the regions currently occupied by the Rüppell's Vultures in Kenya. Of the available 580,367 km^2^ area of Kenya, the model predicted 94.19% as unsuitable (546,683.9 km^2^), 3.69% as an area that is marginally suitable (21,390.3 km^2^), 1.60% as moderately suitable (9306.9 km^2^), 0.48% as a suitable area (2781.7 km^2^) and 0.04% area as an area with high suitability (204.1 km^2^). Most of Kenya (94.19%) can be considered not suitable for Rüppell's vultures. Suitable habitats for the Rüppell's Vultures marked A, B, C, D, and E on the Map of Kenya showing the niche distribution and habitat suitability of Rüppell's vultures (Figure 1b) were found to be next to Masai Mara game reserve, Mount Kenya National Park, Nairobi National Park, Tsavo East National Park and within Hell's Gate National Park (Figure 1c). Most suitable habitats are outside the protected areas (Figure 1d).

### 3.2 Model Evaluation

The ROC and TSS values were 0.965 and 0.808, respectively. The sensitivity and specificity values of AUC were respectively 92.473 and 88.199, while for TSS our model showed values of 92.473 and 88.346.

### 3.3 Variables Influencing Habitat Suitability for Rüppell's Vulture

**FIGURE 2:**
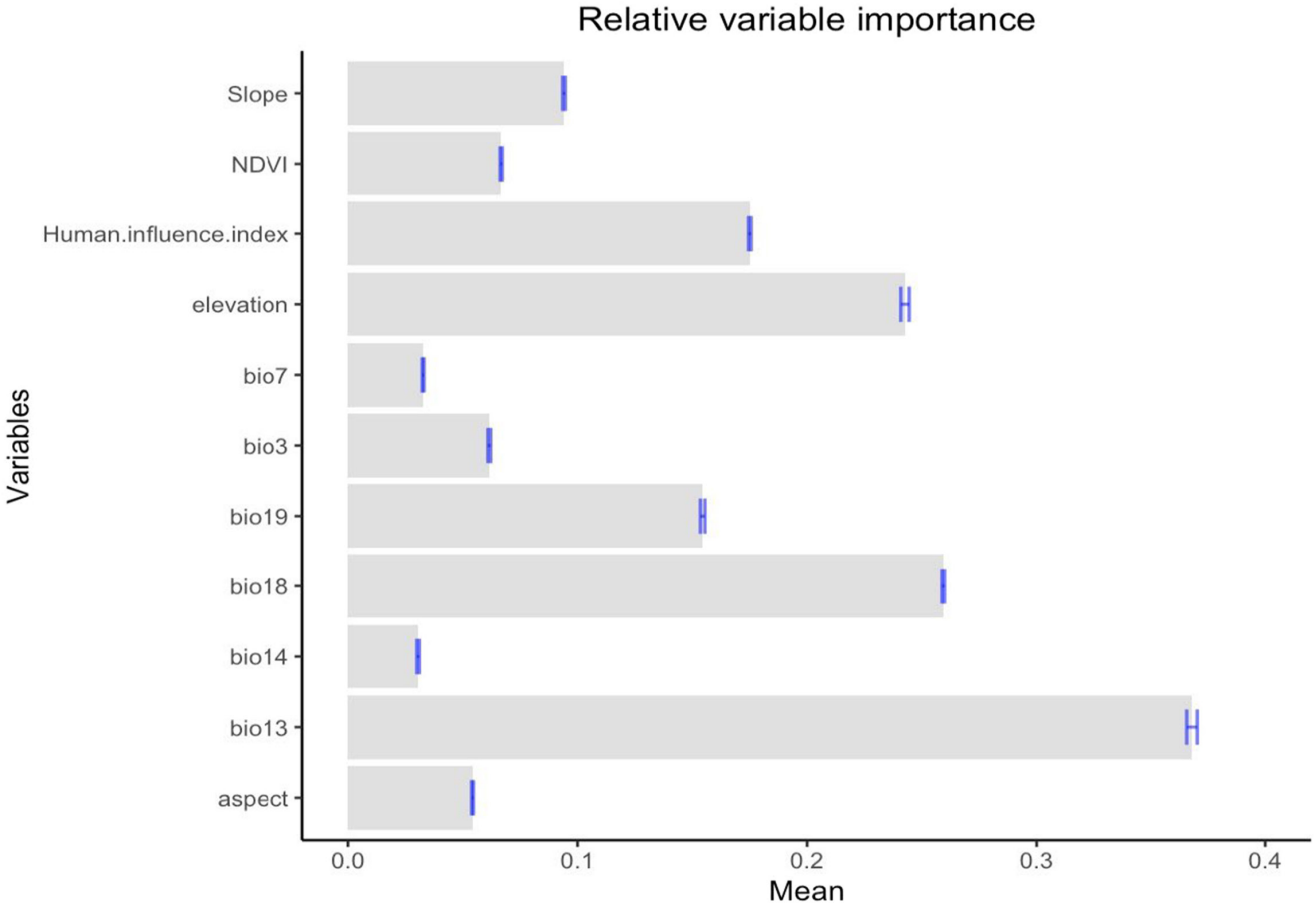
Relative importance of environmental factors to the distribution of Rüppell's Vultures in Kenya.

Precipitation was established to be key in determining the suitability of Rüppell's vulture habitats. Precipitation of the wettest month influenced the distribution of the vulture by 25.1%, and in the warmest month by 18.1%. Precipitation and elevation were the key environmental predictors of habitat suitability and the niche distribution of the Rüppell's vulture. Isothermality (2.4%), Normalized Difference Vegetation (2.4), Aspect (2.2%), Annual temperature range (2%), and precipitation of the driest month (1%) had the least effect on the distribution model (Figure 2).

### 3.4 Response Curves

**FIGURE 3:**
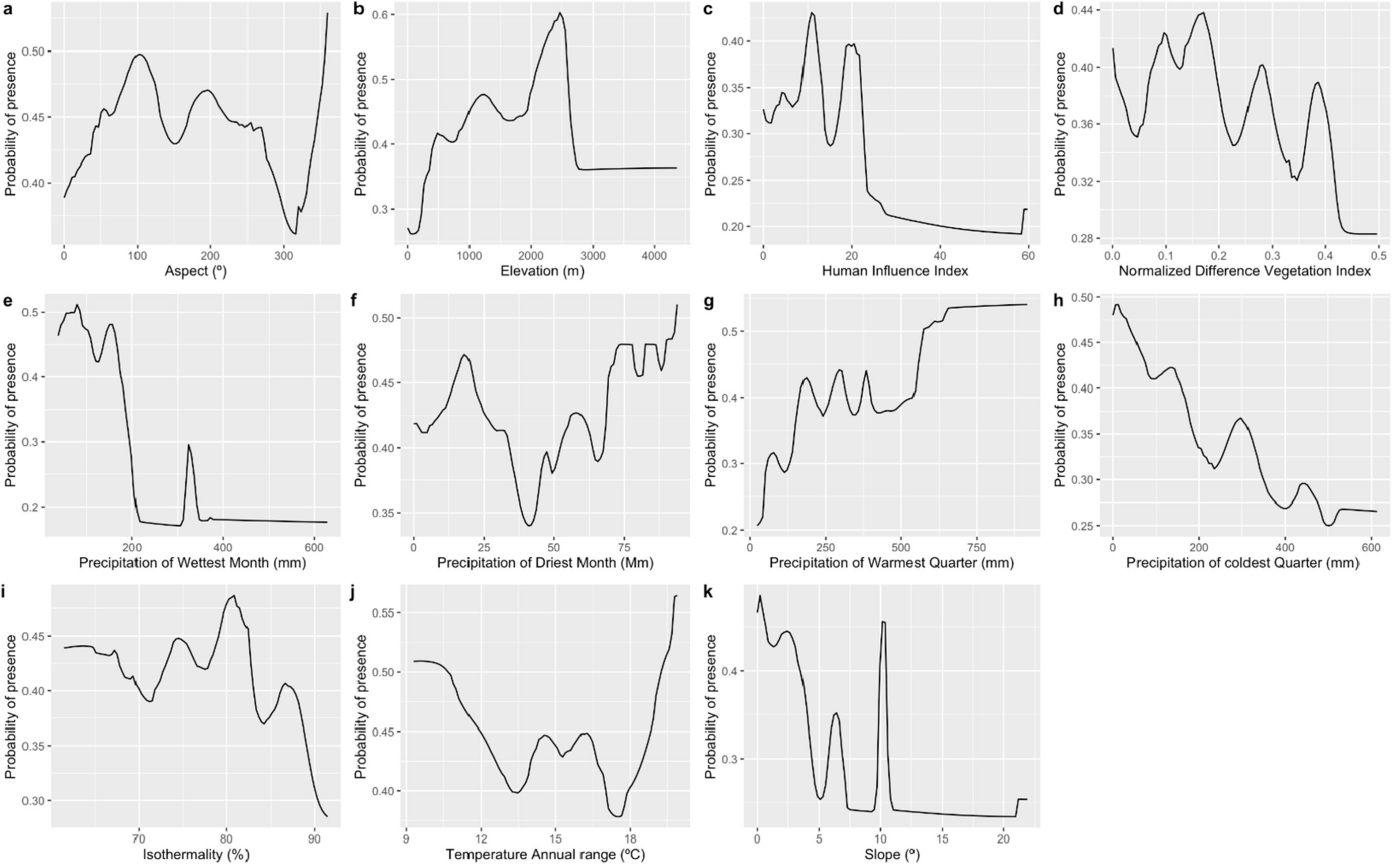
Response curves of 11 environmental variables in Rüppell's Vultures habitat distribution model. Aspect (a), Elevation (b), Human index (c), Normalized Difference Vegetation Index (d), Bio13: Precipitation of Wettest month (e), Bio14: Precipitation of Driest Month (f), Bio18: Precipitation of Warmest Quarter (g), Bio19: Precipitation of Coldest Quarter (h), Bio3: Isothermality (i), Bio7: Temperature Annual range (j), slope (k).

The most important variables for Rüppell's Vultures distribution were precipitation of the wettest month (BIO13), precipitation of the warmest month (BIO18), elevation, Human influence index, Precipitation of the coldest quarter (BIO19). The ideal precipitation for Rüppell's Vultures during the driest month is between 75 and 95 mm, while the optimum precipitation of Rüppell's Vultures during the wettest month ranges between 0 and 200 mm. The distribution of Rüppell's Vultures during the warmest quarter starts to decline when precipitation goes below 125 mm and suitable elevation for the distribution of Rüppell's Vultures is between 1000 and 2500 m. Slope, Human Influence Index, aspect, and isothermality contributed the least to the model (Figure 3).

## 4 Discussion

### 4.1 Suitability Zones for Rüppell's Vultures' Survival

Most areas in Kenya are not suitable for Rüppell's Vultures with a small range of 5.81% being suitable. Areas near protected areas including Masai Mara National Reserve, Hell's Gate National Park, Mount Kenya National Park, Nairobi National Park, and Tsavo East National Park were found to be highly suitable places for the vultures for either scavenging for food or breeding. Given that the Rüppells vulture is a raptor that nests on cliffs, breeding substrate availability around the protected areas is critical for the species' habitat (Mihoub et al. 2014). Additionally, the Gyps vulture favors open territory like in the case of the protected areas to scavenge for food (Dobrev and Popgeorgiev 2021). Similar findings were reported in Spain, where the Griffon vulture was established in the finest rocky surroundings and locations with a lot of livestock (Arkumarev, Dobrev, and Stamenov 2019; Donazar 1990). Highly suitable areas provide favorable conditions for shelter, forage, and water (Jha, Kanaujia, and Jha 2022). Vulture conservation is reliant on safeguarding the colony, mature trees for nesting, breeding, and nesting cliffs, as well as the surrounding habitat in terms of food supply, such as carrions for appropriate foraging. Therefore, it is, imperative to protect the niche areas close to these protected areas to act as conservation buffers for the protection of the species. Such areas, particularly one holding breeding or nesting colonies, could be classified as a Rüppell's Vulture sanctuary, like the case of Hell's Gate National Park. Hell's Gate National Park is the only protected area observed to be holding breeding and nesting colonies for Rüppell's Vultures in Kenya. Suitable habitats of Rüppell's Vultures mostly occurred outside but near the protected areas. This could be the primary cause of vulture reductions throughout Africa (Ogada 2014). Thus, knowing the niche range of vultures necessitates an understanding of the habitat selection process (Mateo‐Tomás and Olea 2011). Therefore, practical conservation measures should be implemented in the most suitable regions. Marginally suitable, moderately suitable, suitable areas, and highly suitable areas should be prioritized in ecosystem management to prevent disruption of nesting, breeding, and territorial expansion activities (factors influencing the presence of endangered species) (Dobrev and Popgeorgiev 2021). By enabling spatially explicit conservation planning choices, the model created in this work could inform the management of breeding habitats in Kenya (Mateo‐Tomás and Olea 2010). However, to achieve a greater scale of conservation of Rüppell's Vultures across the Kenyan landscape, there is a need for conservation actions with a wide geographic reach (Botha et al. 2017; Santangeli et al. 2019), and to lessen hazards to vultures and other species (Santangeli et al. 2019). To identify and manage regional issues, such as conflicts with humans that may be contributing to vulture population loss, it will also be crucial to work closely with stakeholders around the protected areas (Botha et al. 2017; Buechley et al. 2019). An accurate and thorough understanding of a species' geographic distribution is essential for species management and habitat rehabilitation (Kumar and Stohlgren 2009), especially for critically endangered species of conservation importance (Qin et al. 2017). We created the first predicted habitat distribution map for the declining population of Rüppell's vultures in Kenya to inform conservation planning.

### 4.2 Climatic Variables Influencing Habitat Suitability for Rüppell's Vulture

The two bioclimatic variables including precipitation and elevation were found to have a greater influence on Rüppell's Vulture distribution. There is evidence that rainfall patterns impact vulture breeding success (Bridgeford 2003; Virani et al. 2012). Also, the annual temperature range had a significant effect. Temperature fluctuation regulates vulture reproduction, which directly strains the animal (Baldwin 2009; Phipps et al. 2017). The preference for highly elevated areas could be attributed to the ability to limit human impacts to improve nesting efficiency and enhance predator visibility (Donazar, Hiraldo, and Bustamante 1993; Yamac 2007). The average rainfall around Masai Mara National Park is about 650 mm in the southeast to about 1300 mm in the northwest (Bartzke et al. 2018), Nairobi National Park's annual rainfall increases from 500 mm in the southeast to 800 mm in the northwest (Matiko 2014), and around Mount Kenya National Park average annual rainfall amounts from 1600 to 2000 mm, in Hell's Gate National Park, the average annual rainfall is about 670 mm (Odongo et al. 2015; Willkomm, Vierneisel, and Dannenberg 2016) which all fall within the precipitation suitability range for the vulture. According to Virani et al. (2012), precipitation of 600–1600 mm is most suitable for nesting and breeding of Rüppell's Vulture. The annual rainfall around Tsavo National Park to the western part is around 450 and 350 mm to the east part (Tolvanen 2004), Elevation can also alter how Rüppell's Vulture is dispersed by changing the availability of their food supply (Virani et al. 2011).

### 4.3 Modeling Limitations and Potential Improvements

It is crucial to recognize that where Rüppell's vultures live and how well they adapt are affected by different environmental factors, not just ecological conditions. The study's species distribution models only considered climatic variables, potentially overlooking the intricate interaction of other important factors that influence the distribution of Rüppell's vultures. Our research reveals that the modeling framework we have outlined and applied to Rüppell's vultures offers a versatile tool capable of leveraging the increasing volumes of citizen science data available to produce valuable insights into species distributions. Importantly, it identifies areas where additional survey efforts are needed to enhance confidence in predictions. Originally, citizen science data became popular as a way to study where different species are found, especially when it is hard to use standard ways of collecting samples (Mori et al. 2019; Van Strien, Van Swaay, and Termaat 2013). But even though they gather lots of data about where species are, citizen science datasets often have errors and differences (Kelling et al. 2015). Also, the data available online usually do not give all the details about where samples were taken, including places where the species being studied is not found, and they do not say how much effort was put into finding the species. Without this crucial information, it is difficult to ascertain whether the species is absent or went undetected due to inadequate search efforts (Croft et al. 2019). While these issues make it tough to use citizen science data for developing species distribution models, we believe there is still a lot of useful information in these datasets that deserves careful study and reevaluation for integration in future models.

Integrating all citizen science data related to Rüppell's vulture across major online biodiversity platforms like eBird and iNaturalist, in addition to GBIF, holds significant potential for improving conservation efforts and ecological research. While GBIF provides a valuable global repository, expanding data collection to platforms like eBird and iNaturalist can greatly enhance the coverage, accuracy, and resolution of vulture distribution data. These platforms attract a diverse, engaged user base, leading to more frequent and geographically widespread observations. By consolidating data from multiple sources, researchers can gain a more comprehensive understanding of vulture populations, migration patterns, and habitat use, which is critical for informed conservation strategies.

Moreover, platforms like eBird and iNaturalist offer tools for real‐time data submission and validation, which can help in quickly identifying and responding to emerging threats to Rüppell's vulture populations. According to Matutini et al. (2021), integrating citizen science data from various platforms can significantly improve the quality and utility of biodiversity data, making it more accessible for both researchers and policymakers. Milanesi, Mori, and Menchetti (2020) also emphasize the importance of using diverse data sources to refine species distribution models, particularly for wide‐ranging and threatened species like Rüppell's vultures. Therefore, leveraging the strengths of multiple citizen science platforms can lead to more robust and actionable insights into the conservation needs of these vital scavengers.

When modeling the distribution of Rüppell's vultures, land cover variables can present several limitations despite their importance. Land cover data often comes in broad categories and coarse resolutions, which may not accurately capture the specific habitat features or microhabitats that these vultures depend on. Additionally, there can be a temporal mismatch, as land cover data might not reflect recent environmental changes, leading to potential inaccuracies in the model. The availability of food sources, which are crucial for vultures, is not directly represented by land cover data and can vary greatly depending on other factors like livestock density or human activities. Furthermore, land cover maps may not effectively capture edge effects, landscape fragmentation, or the spatial configuration of different land cover types, all of which can influence vulture behavior.

Imperfect detection due to human observation processes can complicate modeling the distribution of Rüppell's vultures. While site occupancy models address this issue by estimating the probability of both occupancy and detection, they may not be well‐suited for Rüppell's vultures. This species' wide‐ranging, low‐density nature and variability in presence make it challenging to meet the assumptions of these models. Therefore, alternative approaches that better account for vultures' mobility and observation variability might be more appropriate for accurate distribution modeling.

## 5 Conclusion

Rüppell's vulture has a limited niche range in Kenya restricted to the designated protected areas including Masai Mara game reserve, Mount Kenya National Park, Nairobi National Park, Tsavo East National Park, and within Hell's Gate National Park. However, suitable habitats for Rüppell's vulture lie outside most of the protected areas except Hell's Gate National Park.

Precipitation and elevation are important environmental predictors of habitat suitability and niche distribution of the Rüppell's vulture. However, elevation at a local scale is most important for their nesting success.

We recommend conservation policymakers and species protection working groups review conservation management plans to designate Rüppell's vulture suitable habitats in Masai Mara and Hell's Gate National Park as sanctuaries and to map for protection the suitable habitats around Mount Kenya National Park, Nairobi National Park, and Tsavo East National Park through a conservation buffer program to enhance the conservation importance of the protected areas to the critically endangered vulture species.

### 5.1 Recommendations

We recommend increased conservation programs to protect the habitats where the niche of Rüppell's Vultures occurs near protected areas as sanctuaries to encourage enough conditions for the utilization of the species. Hell's Gate National Park is the only park where the niche occurs within the protected area, and we propose the gazettement of the park as an area of conservation interest for the Critically Endangered Rüppell's Vultures. High‐priority protection should be provided to the highly suitable regions around the protected areas as sanctuaries. Also, monitoring and restoring dwindling populations of Rüppell's vultures in their native environment, mapping, and predicting possibly appropriate habitats for vulnerable and critically endangered Rüppell's vultures are essential.

## Author Contributions

**Purity Chepkirui:** conceptualization (lead), data curation (lead), formal analysis (lead), methodology (lead), resources (lead), writing – original draft (lead). **David Chiawo:** conceptualization (supporting), funding acquisition (lead), supervision (lead), writing – original draft (supporting). **Jumbe James:** conceptualization (supporting), methodology (supporting), supervision (supporting). **Simbauni Jemimah:** conceptualization (supporting), methodology (supporting), supervision (supporting). **Jane Mugo:** formal analysis (supporting), methodology (supporting). **Peggy Mutheu Ngila:** methodology (supporting), writing – review and editing (supporting). **Elizabeth Ellwood:** funding acquisition (supporting).

## Conflicts of Interest

The authors declare no conflicts of interest.

## Supporting information


Data S1:

## Data Availability

Included as a Supporting Information.
