# Supplementary material for: Niche Distribution Pattern of Rüppell's Vulture (Gyps rueppellii) and Conservation Implication in Kenya
Source: Ecol Evol. 2024 Dec 6;14(12):e70371. doi: 10.1002/ece3.70371 (PMC11621865; doi:10.1002/ece3.70371)
Supplement: Supplementary file 1 — Data S1: [file ECE3-14-e70371-s001.zip › SUPPORTING MATERIAL.docx]

Data availability

1. Code <https://github.com/purychep/Ruppell-Vultures/blob/main/Purity's%20Code.R>
2. Gbif <https://doi.org/10.15468/dl.wh4ee7>
